# Functional Antibody Responses Following Allogeneic Stem Cell Transplantation for *TP53* Mutant pre-B-ALL in a Patient With X-Linked Agammaglobulinemia

**DOI:** 10.3389/fimmu.2019.00895

**Published:** 2019-04-26

**Authors:** Menno C. van Zelm, Marsus Pumar, Peter Shuttleworth, Pei M. Aui, Joanne M. Smart, Andrew Grigg, Julian J. Bosco

**Affiliations:** ^1^Department of Immunology and Pathology, Central Clinical School, Monash University, Melbourne, VIC, Australia; ^2^Allergy, Asthma and Clinical Immunology Service, Department of Respiratory, Allergy and Clinical Immunology Research, Central Clinical School, The Alfred Hospital, Melbourne, VIC, Australia; ^3^The Jeffrey Modell Diagnostic and Research Centre for Primary Immunodeficiencies, Melbourne, VIC, Australia; ^4^Department of Clinical Haematology and Olivia Newton John Cancer Research Institute, Austin Health, Melbourne, VIC, Australia; ^5^Department of Allergy and Immunology, Royal Children's Hospital, Melbourne, VIC, Australia

**Keywords:** X-linked agammaglobulinemia, allogeneic stem cell transplantation, pre-B-ALL, BTK, IgG, vaccination response

## Abstract

Patients with X-linked agammaglobulinemia (XLA) have failure of B-cell development with lack of immunoglobulin (Ig) production. While immunoglobulin replacement therapy (IgRT) is beneficial, XLA patients remain at risk for infections, structural lung damage, and rarely, neoplasia. Allogeneic stem cell transplantation (alloSCT) may offer a potential cure, but is associated with significant life-threatening complications. Here, we present a 25-year old XLA patient who developed pre-B acute lymphocytic leukemia (ALL) with somatic *TP53* mutation, and treatment for this high-risk malignancy involved full myeloablative conditioning and a HLA-matched sibling alloSCT. Full donor chimerism was achieved for CD3+ and CD3- cell fractions. The patient remains in morphological and flow cytometric remission 14 months post-transplant, with late-onset oral GvHD requiring low dose prednisolone and cyclosporin. Following IgRT discontinuation at 4 months post-transplantation, humoral immunity was established within 14 months as reflected by normal numbers of total B cells, memory B cells, serum IgG, IgM, and IgA, and production of specific IgG responses to Prevenar-13 vaccination. This is only the second reported case of an XLA patient with pre-B-ALL, and the most detailed report of engraftment following alloSCT in XLA. Together with the two previous XLA cases treated with alloSCT, our report provides evidence for the potential for successful humoral reconstitution with alloSCT in patients with B-cell intrinsic antibody deficiency. These observations may be relevant given IgRT, while beneficial, remains an imperfect solution to long-term infectious complications.

## Introduction

X-linked agammaglobulinemia (XLA) is the prototypical form of antibody deficiency with patients suffering from impaired humoral immunity due to a complete lack of antibodies (1). In the early 1990s, the gene affected was found to be a protein tyrosine kinase and named Bruton's tyrosine kinase (BTK) (2, 3). Over the past decades more than 600 unique mutations in BTK have been reported. Although BTK is expressed in both the B cell and monocyte lineages (4), genetic deficiencies specifically abrogate early B-cell development in the bone marrow, whereas monocyte development is normal. Thus, BTK deficiency results in absence of mature B cells in blood and secondary lymphoid organs, and agammaglobulinemia. Since the early 1950s, IgG replacement therapy (IgRT) and antibiotics have been used to prevent and treat infections (1, 5). Despite a significant reduction in infection rate with IgRT from early childhood (6), XLA patients continue to suffer from recurrent sinopulmonary infections, higher rates of chronic lung disease and progressive impairment of lung function during early adulthood (7, 8).

Allogeneic stem cell transplantation (alloSCT) is a potentially curative option, with but poses potentially life-threatening risks, and has rarely been studied in XLA patients (9). Initial attempts of alloSCT without conditioning resulted in failed engraftment in 6 XLA patients (10). Since then, two XLA patients were treated with allograft with conditioning: full myeloablation in a 13 yr-old XLA with acute myeloid leukemia (11), and reduced conditioning in 28 yr-old XLA with recurrent serious infections in spite of IgRT (12). Both cases demonstrated alloSCT provided at least partial reconstitution of antibody levels and responses. Here, we present a detailed report on an adult XLA patient with high-risk pre-B-ALL treated with alloSCT with successful reconstitution of B-cells, serum Igs and post-vaccination specific antibody responses.

## Methods

### Ethics

Diagnostic work-up of blood and research studies including genetics of the patient were carried out with approval of Human Research Ethics committees of The Royal Children's Hospital, The Austin Hospital and The Alfred Hospital (Study 109/15) and after written informed consent was obtained. In addition, the patient has provided written consent for publication of the case report. Data from healthy controls were collected after written consent was obtained and with approval of the human ethics committee of Monash University (Study CF15/771). All studies were performed in accordance with the Declaration of Helsinki.

### Blood Sample Processing and Flow Cytometry

For genetic confirmation of XLA, venous blood was obtained from the patient and processed within 24 h. Two milliliters of blood was set aside for flow cytometry (see below), and the remaining was separated on Ficoll-Hypaque to obtain PBMC which were live frozen in liquid nitrogen for later use. The post-Ficoll granulocytes were lysed with Zap-oglobin II (Beckman Coulter) and genomic DNA was isolated from the remaining nuclei using a GenElute genomic DNA isolation kit (Sigma-Aldrich).

Whole blood was used for determining the absolute counts of CD3+, CD4+, and CD8+ T cells, CD19+ B cells, and CD16+/CD56+ natural killer cells with a diagnostic lyse-no-wash protocol by using commercial Trucount tubes (BD Biosciences, San Jose, CA). In addition, post alloSCT, detailed 10-color flow cytometry of B cells was performed following red blood cell lysis with NH_4_Cl on 1–2 million nucleated cells.

For detection of intracellular BTK, live PBMC were thawed, washed and stained with CD3-FITC (UCHT1), CD20-BV605 (2H7; both from BD Biosciences). Following fixation and permeabilization, cytoplasmic BTK was stained with BTK-PE (53/BTK; BD Biosciences) according to manufacturer's instructions. After preparation, cells were measured on 4-laser LSRII or LSRFortessa flow cytometer (BD Biosciences) in the AMREP flow core facility by using standardized settings (13). Data were analyzed with FACSDiva (V8.0; BD Biosciences) and FlowJo software (v10).

### Sequence Analysis of *BTK* Gene and Transcript

Genomic DNA from post-Ficoll granulocytes was subjected to PCR-amplification of exons 1–19 of the *BTK* gene were using previously published primers (14) containing M13 tails, and products were sequenced with M13 primers by the Micromon facility of Monash University on an Applied Biosystems 3730s DNA Analyzer (Thermo Fisher). Obtained sequences were aligned with the reference sequence from Ensembl using CLC Main Workbench 7 software.

RNA was isolated from post-Ficoll mononuclear cells of the patient with a GenElute mammalian RNA kit (Sigma-Aldrich) and reverse transcribed to cDNA with random primers (Life technologies). Splicing of *BTK* exon 18 was examined through PCR amplification and sequence analysis as above of a 318 bp fragment amplified with a forward primer in exon 17 (5′- ATAGCAAGTTCAGCAGCAAAT-3′) and a reverse primer in exon 19 (5′- TTGGGGCTTGTGGAGAAGAGA-3′).

### Diagnostics of Leukemia and Minimal Residual Disease (MRD)

Flow cytometric immunophenotyping of bone marrow was performed with a leukemia panel at Austin Hospital Pathology, consisting of 12 stains (Supplementary Table 1) with up to 5 fluorescent parameters and acquired on a Navios Flow Cytometer (Beckmann Coulter). Flow cytometric MRD analysis was performed using markers that defined the pre-B-ALL phenotype at diagnosis, i.e., CD45+, CD34+, CD56-, CD19+, CD20+.

Molecular analysis of the tumor sample at diagnosis included Fluorescence *in situ* Hybridization (FISH) of 200 cells with the XL BCR/ABL1/ASS duo fusion translocation probe (Metasystems). Karyotyping was performed and 12 normal metaphase spreads were analyzed. Genomic DNA samples of the tumor at diagnosis and of a skin biopsy of the patient were subjected to a custom-designed myeloid amplicon gene panel (Myeloid v5.4) and sequenced on the Illumina MiSeq using MiSeq v2 chemistry and of a skin biopsy of the patient.

Chimerism analysis was performed by the Bone Marrow Transplant Service of Melbourne Health. CD3-positive and CD3-negative fractions were obtained from blood samples and subjected to fragment analysis and capillary electrophoresis of short tandem repeats with germline DNA of the donor and the recipient as controls (15, 16).

### Diagnostic Measurements of Serum Ig Levels and Responses to Vaccinations

IgG, IgA, and IgM serum levels were measured with an immunoturbidimetric method at Austin Pathology. Following SCT, the patient was revaccinated with Boostrix IPV, Prevenar 13, Hib & menveo, and H-B Vax II. Pneumococcus antibodies to 7/13 serotypes in the Prevenar 13 protein-conjugate vaccine were quantitatively measured by a diagnostic Immunology laboratory at the Royal Children's Hospital, using an in-house validated ELISA according to the WHO methodology.

### Data Analysis and Statistics

All data were analyzed with FACS DIVA v8 and FlowJo v10 software packages. Statistical analysis was performed in GraphPad Prism v7 with the non-parametric Mann–Whitney *U*-test; *p* < 0.05 were considered significant.

## Results

### Clinical Presentation and Genetic Diagnosis of XLA

The patient presented at 16 months old with recurrent episodes of pneumonia and chronic cough. He had panhypogammaglobulinemia and absent peripheral B-cells (< 1%), and was diagnosed with XLA at age 2, and commenced on intravenous IgRT. He received subcutaneous IgRT from age 6 to age 11 due to difficulties with venous access. A chest CT at age 11 confirmed left lower lobe bronchiectasis, subsequently complicated by episodes of hemoptysis, and he underwent a lobectomy at age 17. He was maintained on intravenous IgRT, 33 g every 3 weeks, with maintained IgG trough levels of between 8 and 10 g/L. Despite this, he suffered recurrent conjunctivitis, otitis media and sinusitis and symptomatic bronchiectasis, with colonization by *Haemophilus influenzae*. Prophylactic azithromycin 500 mg 3 days per week was commenced as an adult with symptomatic benefit over 2 years.

**Figure 1:**
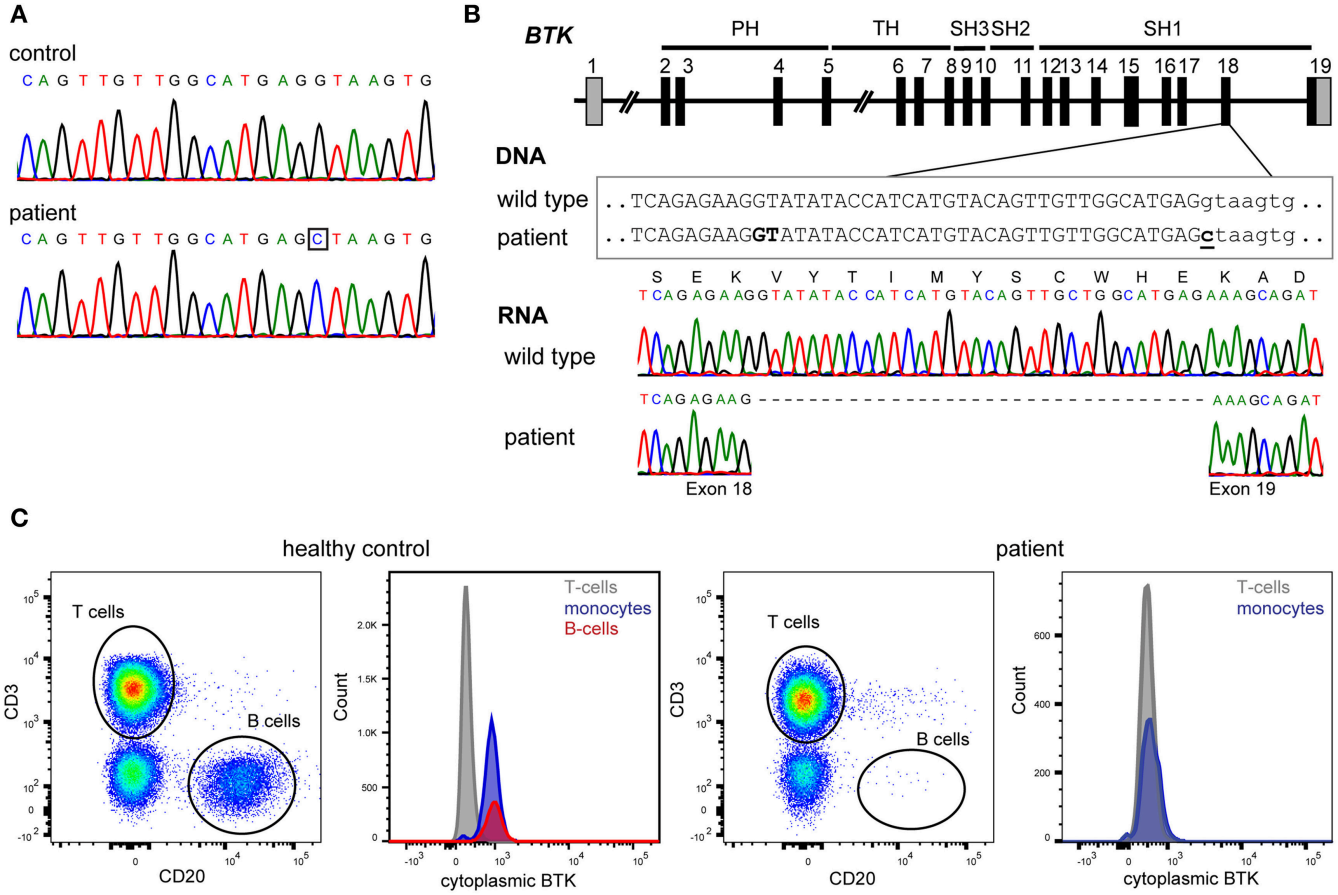
Genetic and protein analysis of BTK. **(A)** Sanger sequencing of all 19 exons and flanking splice sites of *BTK* revealed a hemizygous point mutation. **(B)** The mutation affects the donor splice site of exon 18 (c.1908+1G>C). BTK transcript analysis in blood mononuclear cells revealed the absence of 33 nucleotides at the 3′ end of exon 18, implicating the usage of a cryptic splice site in exon 18, and leading to an in-frame deletion of 11 amino acids (p.Val626_Glu636del). **(C)** The patient's monocytes completely lacked BTK expression as assessed with cytoplasmic staining using flow cytometry with a monoclonal antibody targeting the N-terminal domains of BTK.

At age 24 years, he was documented to have normal number of total T cells (1,497/μl), CD4+ (878/μl) and CD8+ (478/μl) subsets, and NK cells (203/μl), while B-cells remained undetectable. Subsequent sequencing of the *BTK* gene identified a hemizygous c.1908+1G>C splice site mutation downstream of exon 18 (Figure 1A). *BTK* transcript analysis demonstrated the use of a cryptic splice site within exon 18, resulting in an in-frame deletion of 33nt deletion from mRNA (Figure 1B) removing Val626 until Glu636 from the kinase domain of the protein. Intracellular BTK staining in patient's monocytes with a monoclonal antibody raised against the N-terminal domain (clone 53/BTK) revealed a complete absence of protein (Figure 1C).

### Diagnosis and Treatment of pre-B-ALL

Six months after molecular diagnosis at age 24 years, he presented with fevers and weight loss. Blasts were noted on a blood film; a bone marrow aspirate and flow cytometry confirmed the diagnosis of precursor B-cell acute lymphoblastic leukemia (pre-B-ALL): CD19+CD34+TdT+CD10+CD20+sIg- (Supplementary Figure 1). Cytogenetics revealed a complex karyotype with hypodiploidy resulting in near triploidy and mutation analysis demonstrated the presence of the dominant negative TP53 R273H missense mutation at 100% variant frequency (17, 18). The TP53 variant was not present in germline DNA.

Induction therapy with the FRALLE-93 protocol (19) resulted in a suboptimal response with minimal residual disease (MRD) on flow cytometry (7.5% of marrow mononuclear cells). Persistent MRD positivity by flow cytometry (1%) was detected at day 70 after consolidation therapy. Because of the high-risk genotype and MRD positivity, an alloSCT was planned from his HLA-matched, unaffected brother. Salvage treatment with one cycle of blinatumomab (20) resulted in MRD negativity. The allograft proceeded 4 months after diagnosis with conditioning of 60mg/kg etoposide and total body irradiation (TBI) in 11 fractions over 4 days (3 × 3.6 Gy and 1 × 2.4 Gy), and infusion of G-CSF mobilized CD34+ cells (5 × 10^6^ per kg) and GVHD prophylaxis with methotrexate (MTX) and cyclosporine.

**Figure 2:**
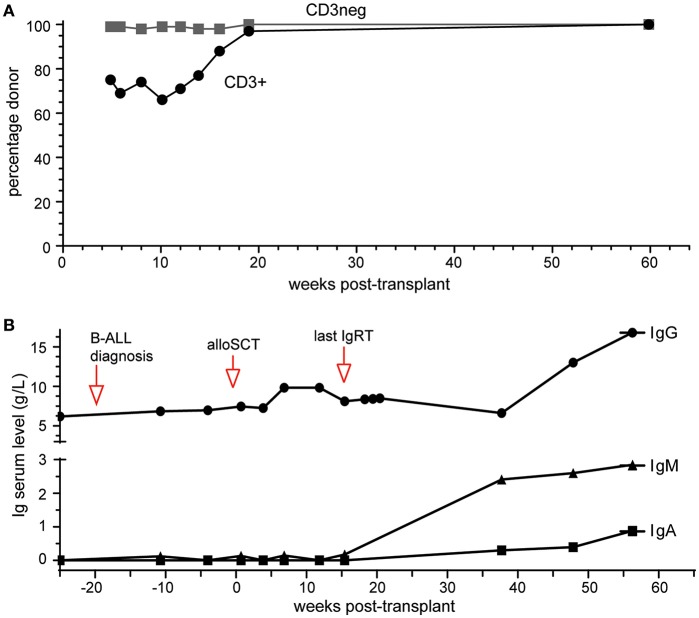
Outcomes of alloSCT. **(A)** Donor chimerism of blood CD3+ and CD3- cell fractions. **(B)** Ig serum levels prior to and following alloSCT. Normal range (in g/L): IgG, 7.0–15.5; IgA, 0.76–3.9; IgM, 0.45–2.3.

Platelet and neutrophil engraftment was noted at day 14 post-transplant and this has been subsequently sustained. Early complete donor myeloid (CD3−) was noted with more delayed but sustained achievement of T lymphoid (CD3+) chimerism (Figure 2A), The patient remains in morphologic and flow cytometry based MRD negative remission 14 months post-transplant, with late-onset limited stage oral GVHD requiring low dose prednisolone and cyclosporin.

### Correction of Antibody Deficiency

IgRT was continued during treatment for pre-B-ALL and initially post allograft, keeping IgG trough levels >6 g/L. It was discontinued 4 months post-transplant with IgG trough levels remaining >6 g/L over the subsequent 10 months (Figure 2B). Serum IgM and IgA were undetectable at 4 months. Serial measurements at 9, 12, and 14 months post-alloSCT demonstrated IgM levels just above the upper limit of normal while serum IgA gradually increased to within normal range (0.88g/L) by 14 months (Figure 2B). Circulating B-cells were first quantified at 9 months post-alloSCT with a value of 1,100 cells/μl, which persisted at the last analysis at 14 months (Table 1). T-cells (both CD4+ and CD8+ subsets) and NK cell numbers were in the low-normal range. Detailed peripheral blood immunophenotyping demonstrated the presence of both naive and memory T cells and B cells, with detectable IgG+ or IgA+ memory B cells (Supplementary Figure 2). The absolute counts of memory B cells were within the normal range, although increased numbers of transitional and naive mature B cells were noted (Table 1). A positive serological response was documented to the protein-conjugated pneumococcal vaccine, Prevenar-13, administered at 9- and 12-months post-transplant (Table 1).

**Table 1:** Lymphocyte subset counts and responses to Prevenar-13 vaccinations post-alloSCT.

**Lymphocyte subset** **(cells/μl)**	**10 months**	**12 months**	**14 months**	**Normal range**
Total T cells	**1,040**	**670**	1,200	1,090–3,020
CD4+ T cells	**420**	**340**	**600**	650–2,000
CD8+ T cells	560	**310**	520	330–1,310
B cells	1,100	1,260	1,166	190–550
Transitional	n.d.	n.d.	130	0.4–29
Naive mature	n.d.	n.d.	970	31–398
CD27+IgD+ memory	n.d.	n.d.	13	3.4–79
CD27+IgD- memory	n.d.	n.d.	36	12–114
NK cells	**50**	**90**	**95**	130–540
**Prevenar-13** **antigens**	**Primary IgG** **(μg/ml)**	**Booster IgG** **(μg/ml)**		**Reference range** **(μg/ml)**
Serotype 4	**0.2**	>3.3		>1.3
Serotype 6B	**<0.1**	>9.1		>1.3
Serotype 9V	**<0.1**	>6.4		>1.3
Serotype 14	**0.9**	1.9		>1.3
Serotype 18C	**0.2**	>7.3		>1.3
Serotype 19F	**0.3**	>14.6		>1.3
Serotype 23F	**0.2**	>6.0		>1.3

*Vaccinations were given at 9- and 12-months post-transplant with evaluation of titers 4 weeks after each. Values below reference range are shown in bold font; values above normal range are underlined. n.d., not determined*.

## Discussion

This case demonstrates successful cure of pre-B-ALL complicating XLA by alloSCT with restoration of B-cell development and functional antibody response.

We are aware of only one previous case of pre-B-ALL in an XLA patient (21), which suggests that human BTK deficiency in itself does not predispose to pre-B-ALL. However, there are data to suggest that BTK may act as a tumor suppressor, and BTK deficiency may predispose to tumor development following a “second hit.” Mice with a genetic deficiency in *Slp65*, a gene encoding an adaptor protein that functions together with BTK, have a block in progenitor B-cell development and spontaneously develop pre-B-cell leukemia. Concomitant deficiency of *Btk* and in *Slp65*-deficient mice enhances development of pre-B-cell leukemia (22). The ALL in our patient contained the dominant negative *TP53* R273H mutation. *TP53* mutations are found in 4% of all pre-B-ALLs and are a known risk factor for therapy resistance (23). Deficiency of *P53* enhances pre-B-ALL formation in *Slp65* deficient mice (24). Although BTK/p53 double knockout mice have not been reported to spontaneously develop leukemia, their B-cells demonstrate both a block in development and an enhanced proliferative capacity (25). It is possible in our patient that the somatic *TP53* mutation in itself was sufficient to lead to a rapidly progressive and chemoresistant ALL. In our case, the germline *BTK* mutation may have a been a contributing predisposing factor, but this association will need to be addressed in future functional studies.

In Australia, XLA patients receive lifelong IgRT but it remains an imperfect solution. On the other hand, alloSCT is not considered a therapeutic option for XLA in Australia due to the recognized severe risks. AlloSCT is standard-of-care in life-threatening forms of immunodeficiency without other treatment options (26), and is being increasingly utilized in children with chronic debilitating, transfusion dependent non-malignant disorders such as thalassemia and sickle cell anemia prior to the development of severe complications (27, 28). While in our case alloSCT was necessary for the B-ALL, an alloSCT may be considered in resource-limited healthcare environments with limited access to IgRT therapy.

Reporting the experience with alloSCT helps provide insights into the benefits and toxicities of this approach as well as transplant management issues including the optimal intensity of conditioning for primary immunodeficiencies. An early series of 6 patients failed to demonstrate engraftment in the absence of myeloablative conditioning (10). Table 2 summarizes 3 subsequent allograft cases, including the patient in this manuscript, in which conditioning resulted in high levels of sustained donor engraftment, restoration of B-cell numbers and detectable antibody levels (11, 12). In Case 1, total Ig levels post-transplant were reported to be normal, as were responses to vaccination, but no details were given for IgA and IgM levels (11). In Case 2 antibody levels were restored, but remained below the range of healthy controls, and an antibody response to vaccination was demonstrated (12). Case 2 had received a mild conditioning regime, which is a potentially lower-risk strategy than the standard conditioning in the other 2 cases. However, it currently remains unclear if the low-dose conditioning impairs immune reconstitution following alloSCT in an adult XLA patient.

**Table 2:** Details of reported cases of XLA with engraftment after alloSCT.

**Report**	**Abu-Arja et al. (11)**	**Ikegame et al. (12)**	**van Zelm et al**.
Case	1	2	3
Age at transplant	13y	28y	25y
Rationale for SCT	AML	Infectious complications	pre-B-ALL
Preconditioning	ETP/CY/ 12Gy TBI	FLU/CY/ATG/ 3Gy TBI	ETP/ 13.2Gy TBI
Donor	Unrelated	Sibling	Sibling
Tissue	PBSC	PBSC	PBSC
GvHD prophylaxis	TAC/MTX	CsA/MMF	CsA/MTX
Donor Chimerism	>95% in leukocytes	100% in T cells and neutrophils	100% in T-cells and non-T cells
Platelet engraftment	n.a.	n.a.	Yes
Neutrophil engraftment	Yes	Yes	Yes
T-cell engraftment	Yes	Yes	Yes
B-cell recovery	Yes	Yes	Yes
Serum Ig recovery	Yes^*^	IgG, IgA, IgM below normal range	IgG, IgA, IgM normal
Specific Ab response	Yes	No	Yes

* No details on individual Ig isotypes provided. n.a., not available.

*AML, acute myeloid leukemia; ATG, rabbit anti-thymocyte globulin; CsA, cyclosporine; CY; cyclophosphamide; ETP, etoposide; FLU, fludarabine; MMF, mycophenolate mofetil; PBSC, peripheral blood-mobilized stem cells; TAC, tacrolimus; TBI, total body irradiation*.

Our case, together with the other two reports, demonstrates that alloSCT with conditioning with full donor chimerism can durably correct the antibody deficiency in patients with XLA and may represent an approach for this disease in patients without significant prior end-stage organ dysfunction.

## Data Availability

All datasets generated for this study are included in the manuscript and/or the Supplementary Files.

## Ethics Statement

Diagnostic work-up of blood and research studies including genetics of the patient were carried out with approval of Human Research Ethics committees of The Royal Children's Hospital, The Austin Hospital and The Alfred Hospital (Study 109/15) and after written informed consent was obtained. In addition, the patient has consented to publication of the case report. Data from healthy controls were collected after written consent was obtained and with approval of the human ethics committee of Monash University (Study CF15/771). All studies were performed in accordance with the Declaration of Helsinki.

## Author Contributions

MvZ and JB designed research. MP, AG, PS, JS, and JB provided clinical care, made treatment decisions and contributed to essential discussions of the study. PA performed experiments. MvZ, AG, and JB wrote the manuscript, and all authors critically read and commented on manuscript drafts and approved of the final version.

### Conflict of Interest Statement

The authors declare that the research was conducted in the absence of any commercial or financial relationships that could be construed as a potential conflict of interest.
