# Supplementary material for: Functional Antibody Responses Following Allogeneic Stem Cell Transplantation for TP53 Mutant pre-B-ALL in a Patient With X-Linked Agammaglobulinemia
Source: Front Immunol. 2019 Apr 26;10:895. doi: 10.3389/fimmu.2019.00895 (PMC6498405; doi:10.3389/fimmu.2019.00895)
Supplement: Supplementary file 1 [file Data_Sheet_1.docx]

**Supplemental Table 1. Flowcytometry panel for leukemia diagnosis**

| Tube | Fluorochrome | | | | |
| --- | --- | --- | --- | --- | --- |
|  | FITC | PE | ECD | PC5.5 | PC7 |
| 1 | CD15 | CD11b | CD16 | CD14 | CD45 |
| 2 | HLA-DR | CD56 | CD34 | CD117 | CD45 |
| 3 | CD7 | CD13 | CD34 | CD33 | CD45 |
| 4 | CD16 | CD56 | CD3 | CD5 | CD45 |
| 5 | CD8 | CD4 | *nil* | CD3 | CD45 |
| 6 | Ig kappa | Ig lambda | CD19 | CD5 | CD45 |
| 7 | CD20 | CD10 | CD19 | CD38 | CD45 |
| 8 | CD65 | CD11c | CD34 | CD25 | CD45 |
| 9 | cyt IgG1a | cyt IgG1a | CD3 | CD22 | CD45 |
| 10 | cyt MPO | cyt CD79a | cyt CD3 | CD117 | CD45 |
| 11 | cyt TdT | CD1a | CD19 | cyt CD22 | CD45 |
| 12 | cyt CD68 | CD9 | CD34 | CD117 | CD45 |

**Supplemental Figure 1**. Immunophenotype of the pre-B-ALL at diagnosis. **A.** Large accumulation of blasts in bone marrow biopsy. **B.** Progenitor-B-cell immunophenotype of blasts. **C.** Detailed characterization of blasts with B-cell markers.

**Supplemental Figure 2.** **B-cell memory reconstitution following alloSCT.** At 14 months post-SCT the patient has small fractions of IgD+CD27+ (1.47%) and IgD-CD27+ (1.64%) memory B cells. The latter express IgG or IgA (1.34%). For comparison, plots from two age-matched males are shown (control 1 and control 2). All plots show total CD19+ lymphocytes with percentages depicted per quadrant.
